# Recent Warming and Cooling in the Antarctic Peninsula Region has Rapid and Large Effects on Lichen Vegetation

**DOI:** 10.1038/s41598-017-05989-4

**Published:** 2017-07-24

**Authors:** Leopoldo G. Sancho, Ana Pintado, Francisco Navarro, Miguel Ramos, Miguel Angel De Pablo, Jose Manuel Blanquer, Jose Raggio, Fernando Valladares, Thomas George Allan Green

**Affiliations:** 1 Departamento de Biología Vegetal II, Facultad de Farmacia, Universidad Complutense, 28040 Madrid, Spain; 2 Departamento de Matemática Aplicada a las TIC, ETSI de Telecomunicación, Universidad Politécnica, 28040 Madrid, Spain; 3 Departamento de Geología, Geografía y Medio Ambiente, Facultad de Biología, Ciencias Ambientales y Química, Universidad de Alcalá, 28871 Alcalá de Henares, Spain; 4 Museo Nacional de Ciencias Naturales, CSIC, 28006 Madrid, Spain

## Abstract

The Antarctic Peninsula has had a globally large increase in mean annual temperature from the 1951 to 1998 followed by a decline that still continues. The challenge is now to unveil whether these recent, complex and somewhat unexpected climatic changes are biologically relevant. We were able to do this by determining the growth of six lichen species on recently deglaciated surfaces over the last 24 years. Between 1991 and 2002, when mean summer temperature (MST) rose by 0.42 °C, five of the six species responded with increased growth. MST declined by 0.58 °C between 2002 and 2015 with most species showing a fall in growth rate and two of which showed a collapse with the loss of large individuals due to a combination of increased snow fall and longer snow cover duration. Increased precipitation can, counter-intuitively, have major negative effects when it falls as snow at cooler temperatures. The recent Antarctic cooling is having easily detectable and deleterious impacts on slow growing and highly stress-tolerant crustose lichens, which are comparable in extent and dynamics, and reverses the gains observed over the previous decades of exceptional warming.

## Introduction

The Antarctic Peninsula is one of the regions on Earth that have experienced some of the largest warming rates during the last decades of the past century, and it has been predicted that this will continue at 0.34 °C per decade until 2100^1^. However, a recent analysis has shown a more complex situation. Mean annual temperatures in the Antarctic Peninsula rose at a rate of 0.32 ± 0.20 °C per decade from 1979 to 1997 and have fallen since then −0.47 ± 0.25 °C per decade from 1998 to 2014^2^.

The single example available at present of vegetation response to increasing temperatures in the Antarctic corresponds to the only two vascular plants found there, *Deschampsia antarctica* and *Colobanthus quitensis*, both of which have shown local increases in populations and possible southward advances^3^. This has raised concerns about possible future colonisation by such species^4^ if temperatures continue to rise as predicted. Manipulation experiments using OTC chambers have reported deleterious effects of both warming and snow accumulation for *Usnea antarctica* ^5^. No report is yet available about the effect of recent cooling on Antarctic vegetation.

Lichens are the dominant vegetation type in the Antarctic Peninsula and adjacent islands with over 400 species present and very high cover in the ice-free coastal areas^6^. However, no studies are available about the effects on lichens of either the 50 years of increasing temperature, or the recent decline in temperature. Monitoring of growth rates is considered the best available indicator of climate change in Antarctica with a 100-fold gradient in rates being found across the continent^7^. That means a dramatic response in annual growth of 30–50%, depending on the species, per 1 °C change in mean temperature. A major effect of warming in the Antarctic Peninsula region is the retreat of glacier fronts^8, 9^ and, as a result, new land surfaces are becoming exposed providing opportunities for lichen colonization^10–13^. Avoiding competition, individuals of each species of these pioneering communities can develop in balance with environmental conditions and can indicate the trend in plant productivity for discrete time intervals over long periods of time.

## Results

**Figure 1:**
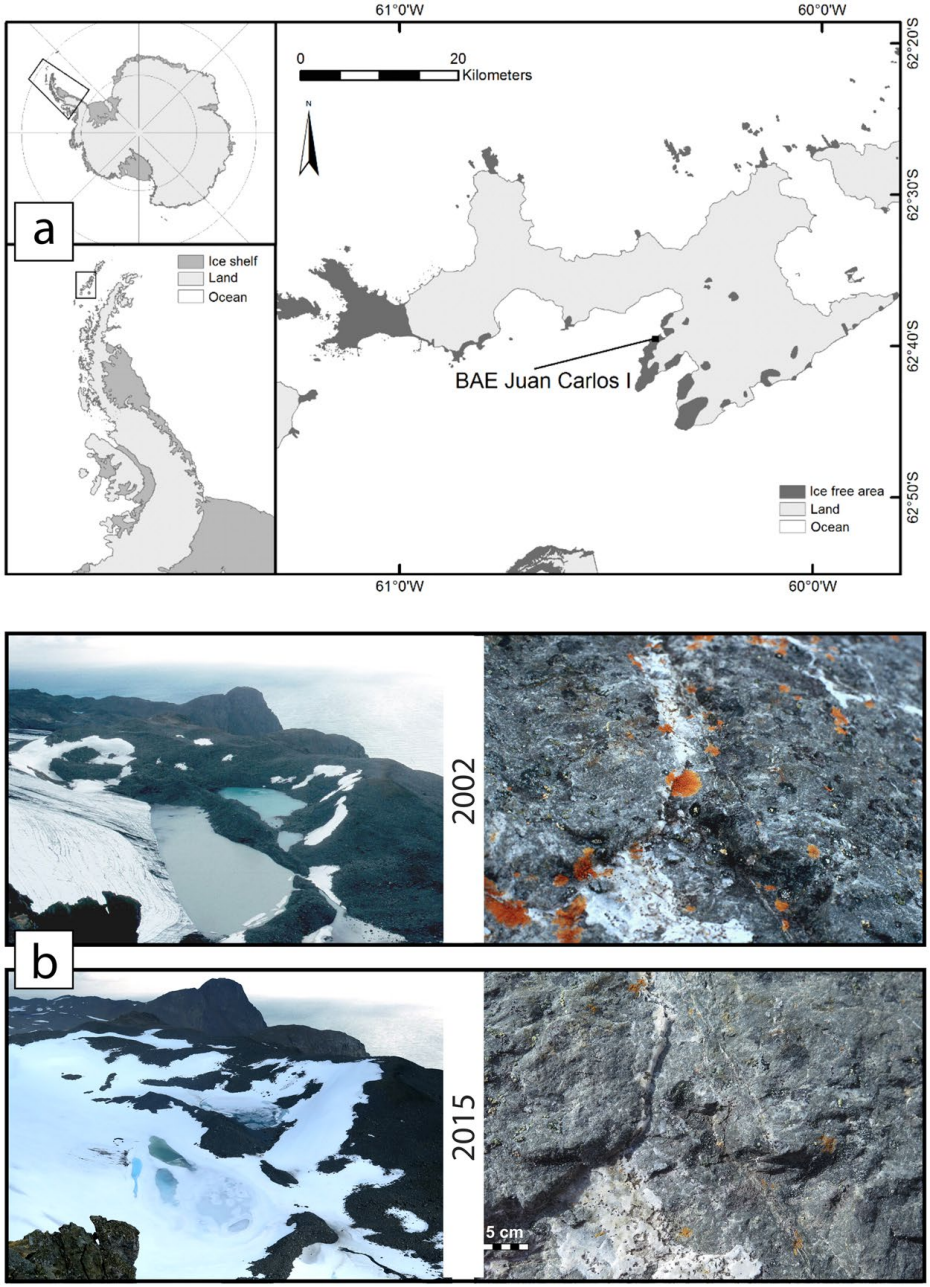
Study site. Location of the moraine under study in Livingston Island, South Shetland Islands, Antarctica (**a**) and photos in January (**b**) of the moraine (left panels) and detail of the lichens (right panel) growing on a boulder in January 2002 (upper panel) and 2015 (lower panel). The map was created using ArcGIS 10.4 ESRI (https://www.arcgis.com).

The lichens studied were located on Livingston Island, South Shetland Islands (Fig. 1a,b), growing on boulders on a moraine formed in 1957^9^. We used standard lichenometric techniques to measure the maximum diameters of the five most common crustose (growing on surface) lichens (*Bellemerea* sp., *Buellia latemarginata*, *Caloplaca sublobulata*, *Rhizocarpon geographicum*, *Acarospora macrocyclos*) and the length of the fruticose (bushy) species *Usnea antarctica*. Measurements were made in 1991, 2002 and 2015 and span a total of 58 years from the exposure of the new rock surface due to glacier retreat giving measurement periods of 34, 11 and 13 years. The measurements taken in 1991 are not a direct calculation of thalli growth rate but an estimation based on the maximum time span for colonization after moraine formation (34 years). Full details are given in the Material and methods section.

### Temperature changes

**Figure 2:**
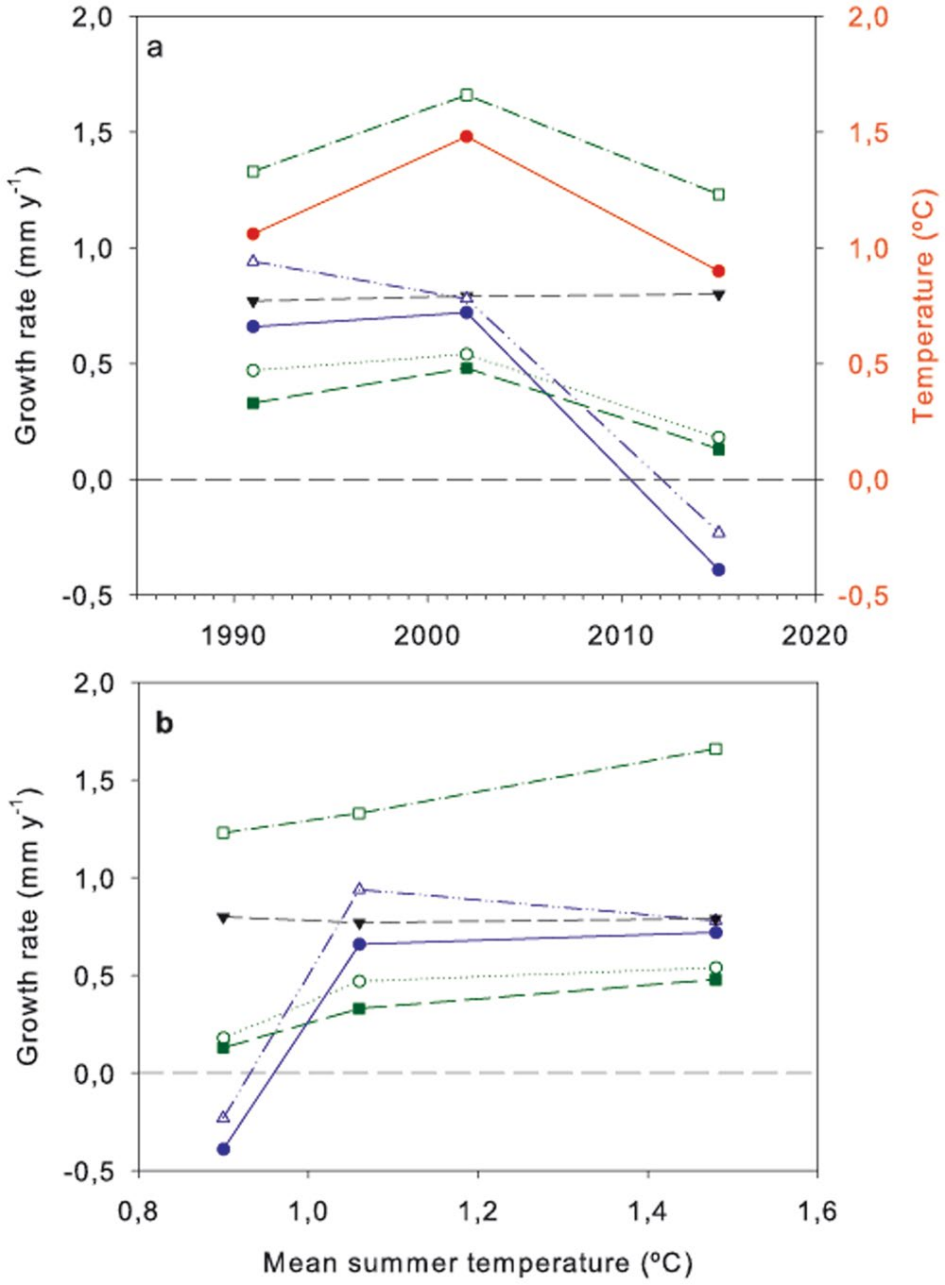
Relationship between annual growth rate and mean summer temperature. (**a**,**b**) Growth rates (mm y^−1^) calculated for the crustose lichens *Acarospora macrocyclos* (), *Bellemerea* sp. (), *Buellia latemarginata* (), *Caloplaca sublobulata* (), *Rhizocarpon geographicum*, () and the fruticose lichen *Usnea antarctica* () from measurements in 1991 (from new surface to 1991, 34 years), 2002 (1991 to 2002, 11 years), and 2015 (2002 to 2015, 13 years) and mean summer (January, February and December) temperatures for the same periods in Bellingshausen Antarctic Base, King George Island () (**a**) and annual growth rates for the same species related to mean summer temperature in Bellingshausen for the same periods (**b**).

The mean summer (December to February, inclusive) temperatures calculated for the three measurement periods were 1.06 °C from the origin of the moraine to 1991 (34 years), 1.48 °C for 1991–2002, and 0.90 °C for 2002–2015 (Fig. 2a). Summer temperatures are used because summer is the main active period for lichens. A complete temperature record was not available for Livingston Island so the temperatures were partly completed using data from Bellingshausen, 62°15′S 58°50′W, on the neighbouring King George Island (Supplementary Fig. 5).

### Lichen growth rates

The six lichen species did not behave similarly and can be classified into three groups based on the pattern of the changes in growth rate and the total range in growth rate (difference between largest and smallest rates) (Fig. 2a, Table 1, Supplementary Fig. 1). The first group contains only *B. latemarginata*, which stands out from the other species by showing almost no change in growth rate. It is the fastest growing crustose lichen with a mean growth rate of 0.79 mm y^−1^ and a small growth rate range of only 0.03 mm y^−1^ (±1.9%). The second group contains three species, the crustose *Bellemerea* sp. and *R. geographicum* together with the fruticose *U*. *antarctica*. All three species show an increase in growth rate from 1991 to 2002 followed by a decline in 2015. The range of growth rates is also very similar at 0.36, 0.35 and 0.40 mm y^−1^, respectively (Table 1; Fig. 2a). The third group contains *C. sublobulata* and *A. macrocyclos*, which have growth rates initially similar to that of *B. latemarginata* with a decrease and increase (respectively) from 1991 to 2002 but followed by a massive decline from 2002 to 2015. The ranges in growth rates are very high: 1.17 and 1.11 mm y^−1^, respectively. The decline is so great that growth rates for 2002–2015 are negative (−0.23 and −0.39 mm y^−1^) and possible causes for this phenomenon are given in the section “Catastrophic change”, see below.

**Table 1:** Growth rates (mm y^−1^) for the 6 measured lichens for the periods 1991 (from new surface to 1991, 34 years), 2002 (1991 to 2002, 11 years), and 2015 (2002 to 2015, 13 years) and mean summer temperatures for the same periods. Right hand column, range of growth rate (minimum to maximum, mm y^−1^) for the 6 studied lichens.

		Growth rates (mm y^−1^)		Growth rate range (mm y^−1^)
Lichen species	**1991**	**2002**	**2015**	
*Acarospora macrocyclos*	0.66	0.72	−0.39	1.11
*Bellemerea* sp.	0.47	0.54	0.18	0.36
*Buellia latemarginata*	0.79	0.77	0.80	0.03
*Caloplaca sublobulata*	0.94	0.78	−0.23	1.17
*Rhizocarpon geographicum*	0.33	0.48	0.13	0.35
*Usnea antarctica*	1.33	1.66	1.23	0.43
Mean summer temperature (°C)	1.06	1.48	0.90	

### Growth rate and temperature

*Usnea antarctica* showed by far the most robust response to mean summer temperature (Fig. 2b, Table 1) with an almost linear response from 1.23 to 1.66 mm y^−1^ (~26%) over a temperature range of 0.58 °C (0.90 to 1.48 °C) and is actually significant (*P* = 0.029) despite the very small data base (only the means were used). When growth rates are normalised to 100% as the fastest rate, then *Belleremea* sp., *R. geographicum* and *A. macrocyclos* show similar responses to temperature but only for 1991 and 2002 (1.06 to 1.48 °C) (Supplementary Fig. 4). There seems to be little doubt that lichen growth is a suitable parameter to follow changes in mean air summer temperature (the growth season) in the absence of any other over-riding factor.

### Catastrophic change

With the exception of *B. latemarginata*, the remaining five species show declines in growth rate between 2002 and 2015, which are much more extreme, with negative growth rates for *A. macrocyclos* and *C. sublobulata*, the third group of lichens (Fig. 2a). Negative growth rates are not possible in crustose lichens, which are firmly embedded in the rock surface, and this massive decline is the result of the disappearance of several large thalli between 2002 and 2015 as visible in photographs (Fig. 1b) and in changes in population structure (Supplementary Figs 2 and 3).

## Discussion

We propose that the most likely causal factors for the loss of some thalli are increased snow fall and longer lasting snow cover, both of which are known to lead to lichen death, the so-called “snowkill”^14^. It is also known that lichens show differential sensitivity to snow burial^15–17^. Snowkill is expected, therefore, to not only lead to the death of some lichens but also to consequential changes in biodiversity because of their different sensitivities.

**Figure 3:**
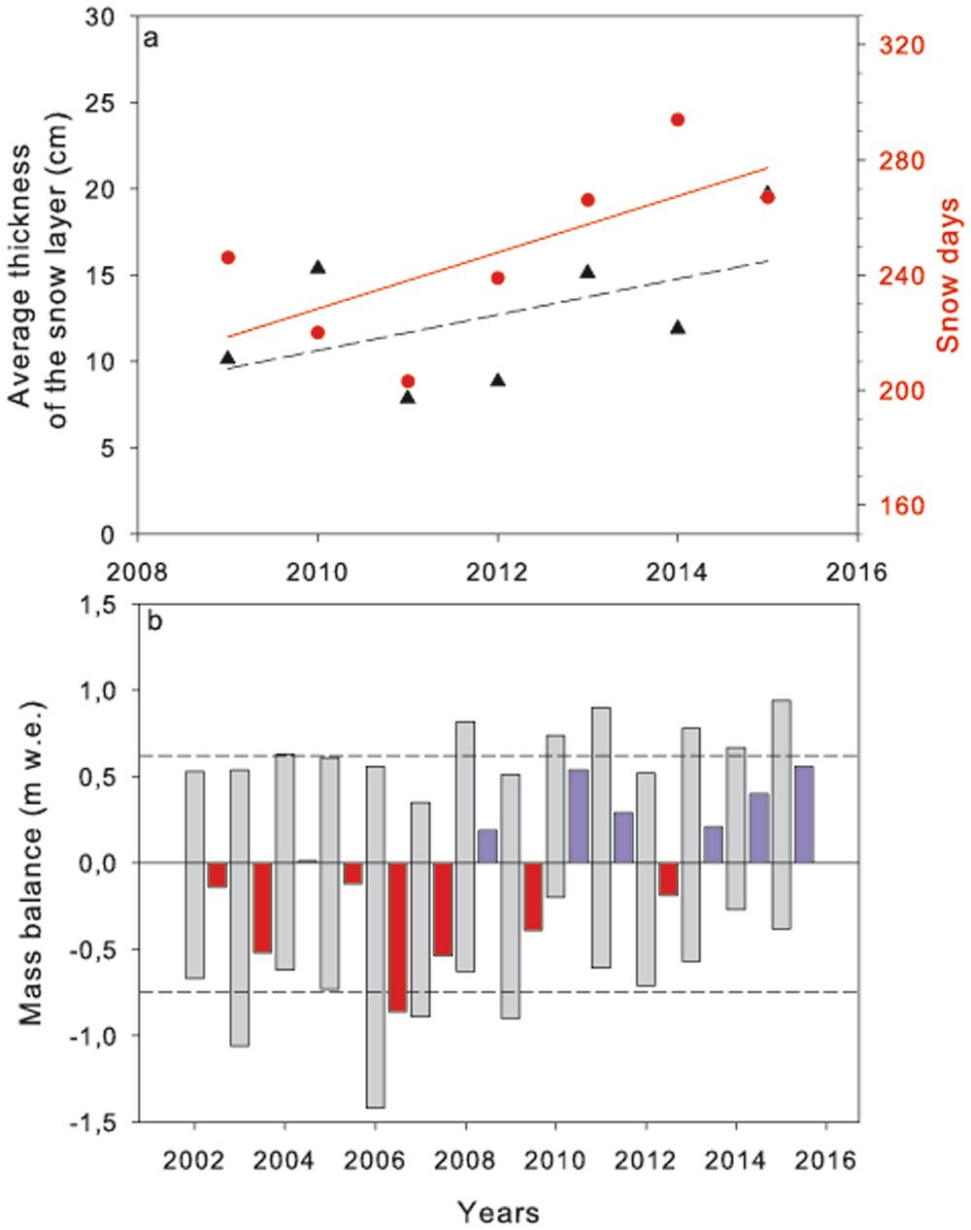
Snow and ice accumulation trends in the last years in the surrounding of the Spanish Antarctic Base on Livingston Island. (**a**,**b**) Average thickness of the snow layer (, r^2^ = 0.278) and number of days with snow (, r^2^ = 0.471) in the last 7 years (**a**) and surface mass balance series of Hurd Glacier (**b**). The grey bars represent the mass balances (positive values, winter balance and negative values, summer balances) and the red/blue bars to their right the resulting annual balances: red if negative (net mass loss), blue if positive (net mass gain). The dashed lines represent the 14-year averages for winter and summer balances. Years shown are southern hemisphere hydrological years.

Several lines of evidence provide strong support for recent increases in snow precipitation and snow cover duration on Livingston Island. Surface mass balance near the studied moraine shows a clear positive trend over the last seven years for both, snow depth and duration of snow cover (Fig. 3a). This has had an effect in the surface mass balance of Hurd Glacier with a shift, in 2007/08, from clearly negative mass balances to predominantly positive mass balances (Fig. 3b). All years with positive mass balance not only have winter balances above the average, indicating that increased snow accumulation has played a key role in the net mass gain, but also summer balances that are less negative and indicate a contribution by lower summer melting. These results agree with long term studies carried out on the South Shetland Islands and in Antarctic Peninsula which, at the beginning of this century, show a break in the positive trends of both snow accumulation and length of the melting season detected in the past decades^18, 19^.

The equilibrium-line altitude (ELA) represents the boundary on a glacier separating the accumulation and ablation zones, i.e. the zones with net mass gains and losses, respectively. At the end of the melting season, the ELA and the snow line altitude are approximately coincident. Although the ELA is actually defined for the glacier surface, it is also a good proxy for the altitude of the snow line on the surrounding areas by the end of the melting season. The moraine under study spans an altitude range of ca. 125–150 m a.s.l., and from Table 2 we can see that for the five hydrological years 2010–2011 and 2013–2015 the ELA was below the altitude of the moraine, indicating that the latter was snow-covered all year round. This is also supported by visual observations and by measurements of snow cover at the end of the melting season in neighbouring mass-balance stakes located at similar and even lower elevations. Until 2002 the research site had been snow free in January when the measurements were made and the glacier fronts were also retreating^9^. However, in 2015 there was an extensive snow cover that was still present in January and this was also the situation in 2016. There are indications showing that the glacier front is once more advancing (Fig. 1b).

**Table 2:** Winter (Bw), summer (Bs) and annual mass balance (Ba) expressed as m w.e. (meter water equivalents) together with the Equilibrium Line Altitude (ELA) calculated in Hurd Glacier for the last 14 hydrological years. Mean and standard deviation are shown at the bottom of the table. For ELA values in bold indicate an ELA below the moraine altitude and therefore continuous snow cover.

Hydrological year	Bw (m w.e.)	Bs (m w.e.)	Ba (m w.e.)	ELA (m a.s.l.)
2002	0.53	−0.67	−0.14	240
2003	0.54	−1.06	−0.52	310
2004	0.63	−0.62	0.01	205
2005	0.61	−0.73	−0.12	235
2006	0.56	−1.42	−0.86	280
2007	0.35	−0.89	−0.54	280
2008	0.82	−0.63	0.19	185
2009	0.51	−0.9	−0.39	250
2010	0.74	−0.2	0.54	**0**
2011	0.90	−0.61	0.29	**130**
2012	0.52	−0.71	−0.19	225
2013	0.78	−0.57	0.21	**95**
2014	0.67	−0.27	0.40	**0**
2015	0.94	−0,38	0.56	**0**
Mean	0.65	−0.69	−0.04	174
Std dev.	0.17	0.32	0.43	110

### *Usneaantarctica*

This species stands out as the lichen that not only has the best relationship between growth rate and mean air summer temperature but also shows no negative effects of increased snow fall (Fig. 2). The species is different to the other five measured because it is fruticose and stands up above the rock surface. It will clearly not be so much affected by lighter snowfalls as the crustose species. The lichen also tends to grow on exposed locations meaning that it is not only less likely to be buried in snow but also can benefit by trapping blowing snow to improve water relations. It has been suggested^5^ that this species is particularly vulnerable to snow cover and increased winter respiration due to higher temperatures, but this is probably a consequence of the use of Open Top Chambers, which not only increase snow depth and duration but also cause a temperature increase. In contrast, under natural conditions, our results suggest that it is actually one of the least affected species.

## Conclusions

Lichens, despite their slow growth rates, quickly track the effects of the complex climatic changes in the maritime Antarctic. Considering only the air temperature, an increase of only 0.58 °C results in a 26% rise in the growth rate of *U. antarctica*, a species whose growth rate appears to almost perfectly track ambient temperature.

The snowkill process observed here as a consequence of the recent cooling of the Antarctic Peninsula is a biologically relevant “tipping point” as, after a gradual decline of lichen growth as the snow cover increases, there is a threshold in the duration of snow cover that leads to lichen death once it is surpassed. However, disaster for some species represents opportunities for other species. Decadal or centurial climatic oscillations, such as those observed in the Antarctic Peninsula, may have initiated rare colonisation events in the Dry Valleys^20^ and explain lichen communities of different ages in Ryder Bay, Adelaide Island^21^ and the Arctic^22^.

Failure to consider interacting environmental factors such as snow cover duration in addition to fluctuations in mean ambient temperature are rendering biased estimations of the ecological impact of climate change. Our study indicates that the positive effects of enhanced annual temperatures on the productivity of Antarctic plant communities and the potential colonizing success of alien species could be overestimated if other interacting factors are not considered.

## Material and Methods

### Lichenometric measurements

The current work is the most recent activity of the monitoring of the colonization process of a moraine close to the Spanish Antarctic Station Juan Carlos I started 24 years ago (Fig. 1). This moraine, which lies in front of a side lobe of Hurd Glacier (Fig. 1), at 125–150 m above sea level, is known to have originated following a glacier retreat event by 1957^23^. The first lichenometric measurements were made in 1991^10^, and growth rates were calculated assuming a maximum period of time for colonization and growth of 34 years. Eleven years later, a new set of measurements was carried out^12^. In February 2015 the moraine was revisited, extending the time span of observations to 58 years. Between these lichenometric studies, photos of the moraine and of the selected boulders were taken nearly every year.

In 1991 ten boulders of different size were marked and the lichens on them measured. We were able to identify these boulders in following years and were able to repeat measurements on the same lichen community. In 2015 we observed that one boulder had disappeared, most probably having fallen down the steep slope of this rather unstable young moraine. Fortunately, none of the largest thalli measured in previous years were growing on this boulder, so that calculations of maximum annual growth rate of the whole community were not affected.

In 1991, we had selected and measured, on each of the boulders the ten largest thalli of the six most abundant lichen species: *Acarospora macrocyclos* Vain., *Bellmerea* sp., *Buellia latemarginata* Darb., *Caloplaca sublobulata* (Nyl.) Zahlbr. *Rhizocarpon geographicum* (L.) DC. and *Usnea antarctica* Du Rietz. In 2015, as in previous years (1991, 2002), we measured the maximum and minimum axes (diameter) of the more or less circular crustose thalli. One species, *U. antarctica*, was fruticose and for these samples we measured the cross section and the height of each thallus. The measurements were made with a calliper with an instrumental error of less than 0.1 mm. At the same time, we carried out an exhaustive floristic survey of all the selected boulders to detect possible changes in the floristic composition of this pioneering lichen community. Comprehensive^24^ and regional^25, 26^ floras were used for the identification of the lichen specimens. Vouchers of all identified taxa are deposited at the herbarium (MAF) of the Botanical Institute of Complutense University, Madrid.

Mean annual growth rate of each species was calculated from the increase in diameter since the previous measurement, using the average of the ten largest thalli^27^.

### Glacier surface mass balance measurements

Information on the surface mass balance (net balance between accumulation and ablation processes; see ref. 28) of a glacier can provide supporting evidence of the recent shift in the local climatic conditions, in particular, for the increase in snow accumulation and the decrease of summer melting. Surface mass balance was calculated by the direct glaciological method^29^ which utilises a combination of field measurements, including accumulation and ablation at a net of stakes deployed on the glacier surface, glacier-wide snow thickness probing, and snow density at snow pits. These measurements have been made since the field season 2001/02 on both Hurd and its neighbouring Johnsons glaciers^30^. The net of stakes covering Hurd and Johnsons glaciers consists of about 50 wooden poles, of 3.65 m in length and 0.045 m in diameter, about half of the total on each glacier. These stakes are measured (GPS positioning and length of stake above the snow/ice surface) at least twice per year, at the beginning and end of the summer season. These stake measurements are complemented with snow thickness measurements by snow probing, to the depth of the last summer layer, at the locations of the stakes plus ca. 50 additional points on the glacier surface (see ref. 30). From this set of measurements we are able to compute point surface mass balances (winter, summer and annual –annual, as net result from winter accumulation minus summer ablation). These point mass balances were then interpolated over the glacier surface using a 25- m resolution grid using kriging interpolation. Balances were calculated in elevation bands of 20 m and then integrated to the full extent of the glacier, thus providing glacier-wide surface mass balances *B*_*w*_, *B*_*s*_ and *B*_*a*_ (*B* denotes glacier-wide mass balance, with subscripts *w*, *s* and *a* used to represent winter, summer and annual values). The Equilibrium Line Altitude (ELA, average altitude of zero mass balance), was calculated in elevation bands of 5 m, as the band with the annual balance closest to zero. The elevation data were provided by a digital elevation model (DEM) of corresponding grid size, obtained from precise geodetic measurements during the field seasons 1999/2000–2000/01. There was no need to update the DEM for subsequent campaigns, because the surface-elevation and front-position changes have been very small during the entire observation period 2001–2015. Mass balance years used throughout this paper are hydrological years for the Southern Hemisphere, so e.g. year 2002 starts on 1 April 2001 and ends on 31 March 2002.

### Snow cover and soil temperature trends

The soil and air thermal behaviour and the snow layer evolution were studied at an experimental site (35 m a.s.l) located near the Spanish Antarctic Station Juan Carlos I. The method to calculate the snow layer evolution uses temperature data acquired, over the period 2009–2015, by an array of temperature sensors (iButton with an accuracy of 0.5 °C) installed above the surface along an isolated (wooden) post^31^ at heights of 5, 10, 20, 40, 80 and 160 cm. This approach to detect the presence of snow cover is based on the differences in temperature at different levels above the surface and the amplitude of the daily temperature variations. When the surface sensor and daily temperature difference is ≤0 °C, it is considered that snow covers the surface. We applied all these methods to obtain a precise determination of the snow cover presence and an approach to the thickness evolution. We calculated the days with snow per season as the number of days between the snow onset and snow offset. First and last day of snow on the ground (‘snow onset’ and ‘snow offset’, respectively) were also detected from the sharp change in the daily variability of ground surface temperature^32^ by means of a metallic plate in contact with the soil that monitored soil surface temperature changes. The plate is buried at about 1 cm to avoid its heating by direct solar radiation. The gradient in soil temperatures from a shallow borehole 2.40 m deep with sensors located at different levels (5, 25, 50, 100, 230 cm) have been recorded at hourly intervals since 2003 in a continuous annual data series. Miniature single-channel data loggers (Tiny Talk, Gemini Co.) with NTC-10K thermistors with a resolution better than 0.05 °C and an accuracy of 0.1–0.2 °C have been used. This allows us to extrapolate the soil surface temperature, *Ts*. From these data we calculated the soil/air freezing and thawing indexes (FDD-soil/air and TDD-soil/air) and the *n*-factor (Supplementary Fig. 6), defined as:

1 $$Ts=T(50\,cm)-2[T(50\,cm)-T(25\,cm)]$$

2 $$FDD/TDD={\int }_{freezing/thawing}(|Tf-Ts|)dt$$

3 $$n-facto{r}_{FDD/TDD}=\frac{FD{D}_{soil}}{FD{D}_{air}}=\frac{TD{D}_{soil}}{TD{D}_{air}}$$

where *T*_*f*_ is the water melting point.

### Meteorological data

Temperature records for Livingston Island are available^33^. As records for Livingston Island weather station (BAE JCI, 62°39′46′′S, 60°23′20′′W) were not complete for the study period we use data from different weather stations in South Shetland Islands published by the British Antarctic Survey web page (https://legacy.bas.ac.uk/met/READER/surface/stationpt.html). Previous studies have shown significant linear relationship between meteorological records obtained from different weather station in South Shetland Islands^34^. Stations that met the requirements of proximity to the study locality and long temperature records were chosen: Teniente R. Marsh Airport, 62°11′S 58°59′W and Bellingshausen, 62°15′S 58°50″W, both on King George Island. Relationships between mean annual and mean summer air temperatures (MAST) and year were investigated with regression analysis using Sigmaplot 11.0 (San José, California, USA) program. Years in which any of its monthly surface temperature percentage of observations are too low to calculate an accurate mean (<90%) have been rejected (this affected years 2010, 2011, 2012 and 2015 at Marsh Station).

#### Statistical approach

Pearson correlations (XLSTAT) were calculated between all lichen species and between lichen species and mean annual temperature for the mean growth rates for the three measurement periods. The small number of samples (for each lichen there were only 3 data points, the 3 mean annual growth rates) meant that statistically significant differences were not anticipated. The aim of the analysis was to discover any possible trends in response for the different lichens (Supplementary Table 1).

## Electronic supplementary material

Supplementary Material
