# Supplementary material for: Recent Warming and Cooling in the Antarctic Peninsula Region has Rapid and Large Effects on Lichen Vegetation
Source: Sci Rep. 2017 Jul 24;7:5689. doi: 10.1038/s41598-017-05989-4 (PMC5524963; doi:10.1038/s41598-017-05989-4)

RECENT WARMING AND COOLING IN THE ANTARCTIC PENINSULA REGION HAS RAPID AND LARGE EFFECTS ON LICHEN VEGETATION

* Leopoldo G. Sancho[1], Ana Pintado[1], Francisco Navarro[2], Miguel Ramos[3], Miguel Angel De Pablo[3], Jose Manuel Blanquer[1], Jose Raggio[1], Fernando Valladares[4], Thomas George Allan Green[1]

[1] Departamento de Biología Vegetal II, Facultad de Farmacia, Universidad Complutense 28040 Madrid, Spain.

[2] Departamento de Matemática Aplicada a las TIC, ETSI de Telecomunicación, Universidad Politécnica, 28040 Madrid, Spain.

[3] Departamento de Geología, Geografía y Medio Ambiente, Facultad de Biología, Ciencias Ambientales y Química, Universidad de Alcalá, 28871 Alcalá de Henares, Spain.

[4] Museo Nacional de Ciencias Naturales, CSIC, 28006 Madrid, Spain.

* Correspondence to sancholg@ucm.es

	Acarospora	*Bellemerea*	*Buellia*	*Caloplaca*	*Rhizocarpon*	*Usnea*
Correlation coefficient (*R*)	0.746	0.830	**-0.998**	0.619	0.944	**0.999**
Probability (*P*)	0.464	0.377	**0.040**	0.575	0.214	**0.029**

Supplementary Table 1. Correlation coefficients and probabilities for the comparison between mean summer temperature for the three time periods and the growth rate of the 6 lichens (XLStat, significant results are bolded).

Supplementary Fig 1.: *Bellmerea* sp (1),. *Buellia latemarginata* (2) and *Rhizocarpon geographicum* (3) in 1991. 2002 and 2015 (scale = 1 cm and is identical for all pictures).

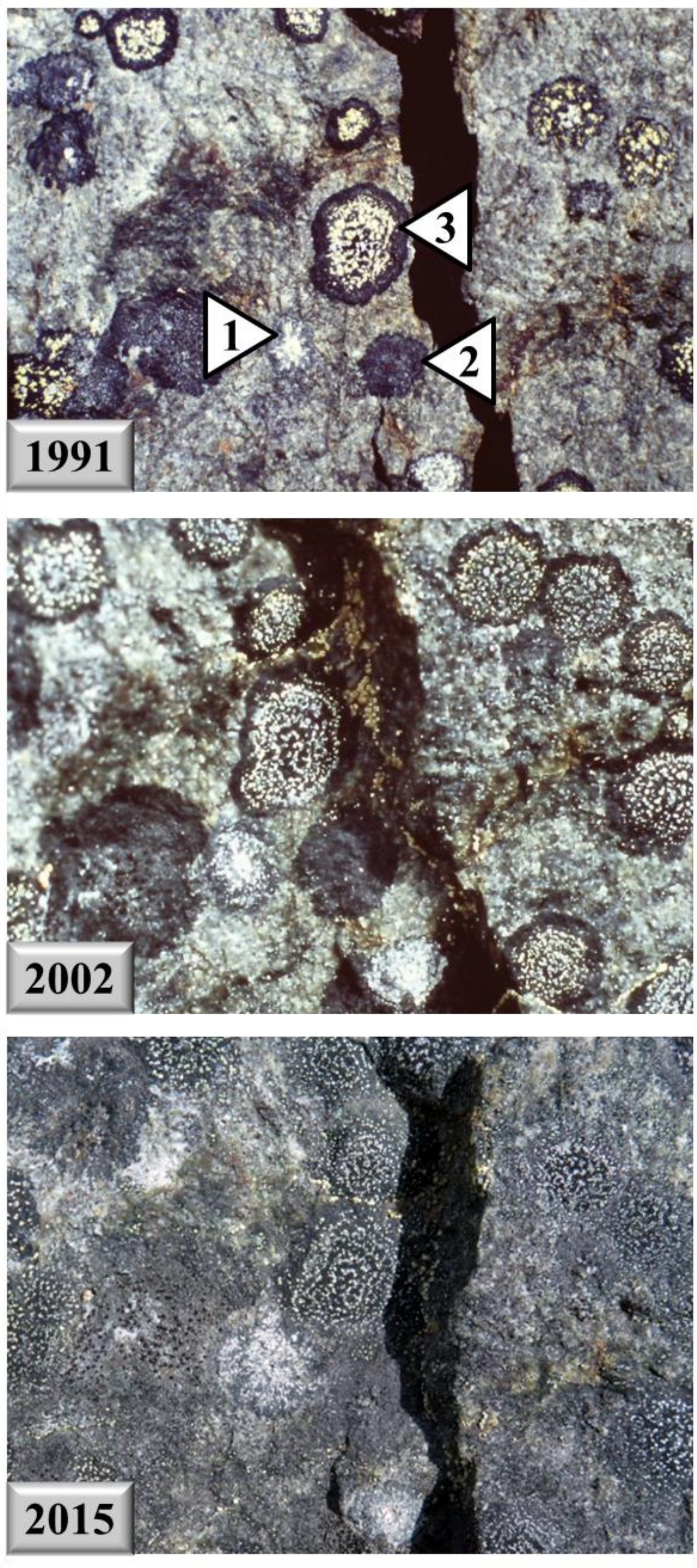

Supplementary Fig. 2: Population development of the six selected species. Y axis is minimum diameter and X axis is maximal diameter (mm); black circle 1991, white circle 2002, grey circle 2015.

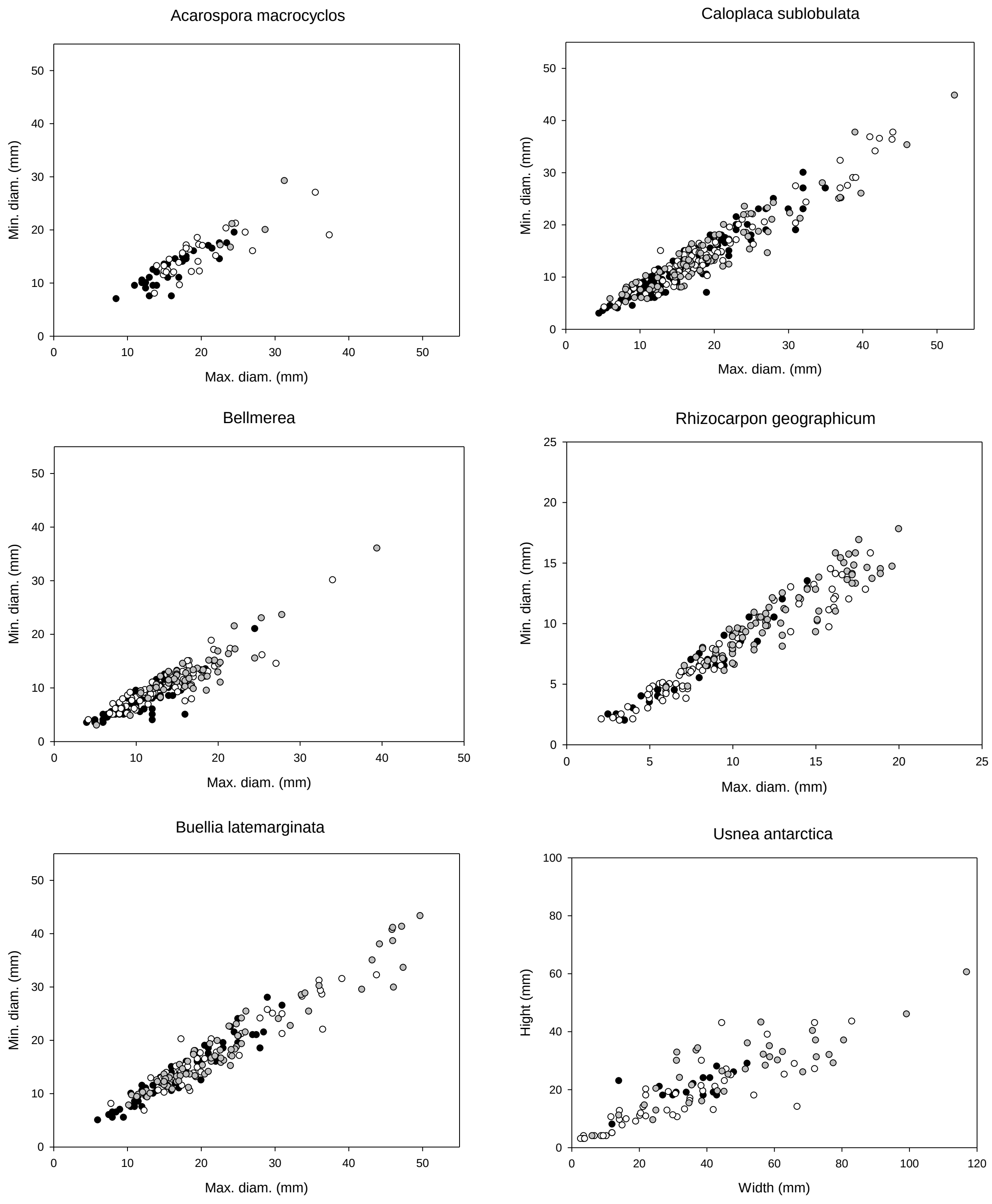

Supplementary Fig. 3: largest diameter for *Caloplaca sublobulata* (mm, the 8 largest thalli, upper panel) and longest length for *Usnea antarctica* (mm, 10 longest thalli, lower panel), measured in 1991, 2002 and 2015. The dashed lines for *C. sublobulata* mark the largest and smallest of the 6 thalli in 2002. For *U. antarctica* the continuous line joins the mean values for the 3 measurements whilst the dashed line shows the expected change if the growth rate between 1991 and 2002 was then maintained until 2015.

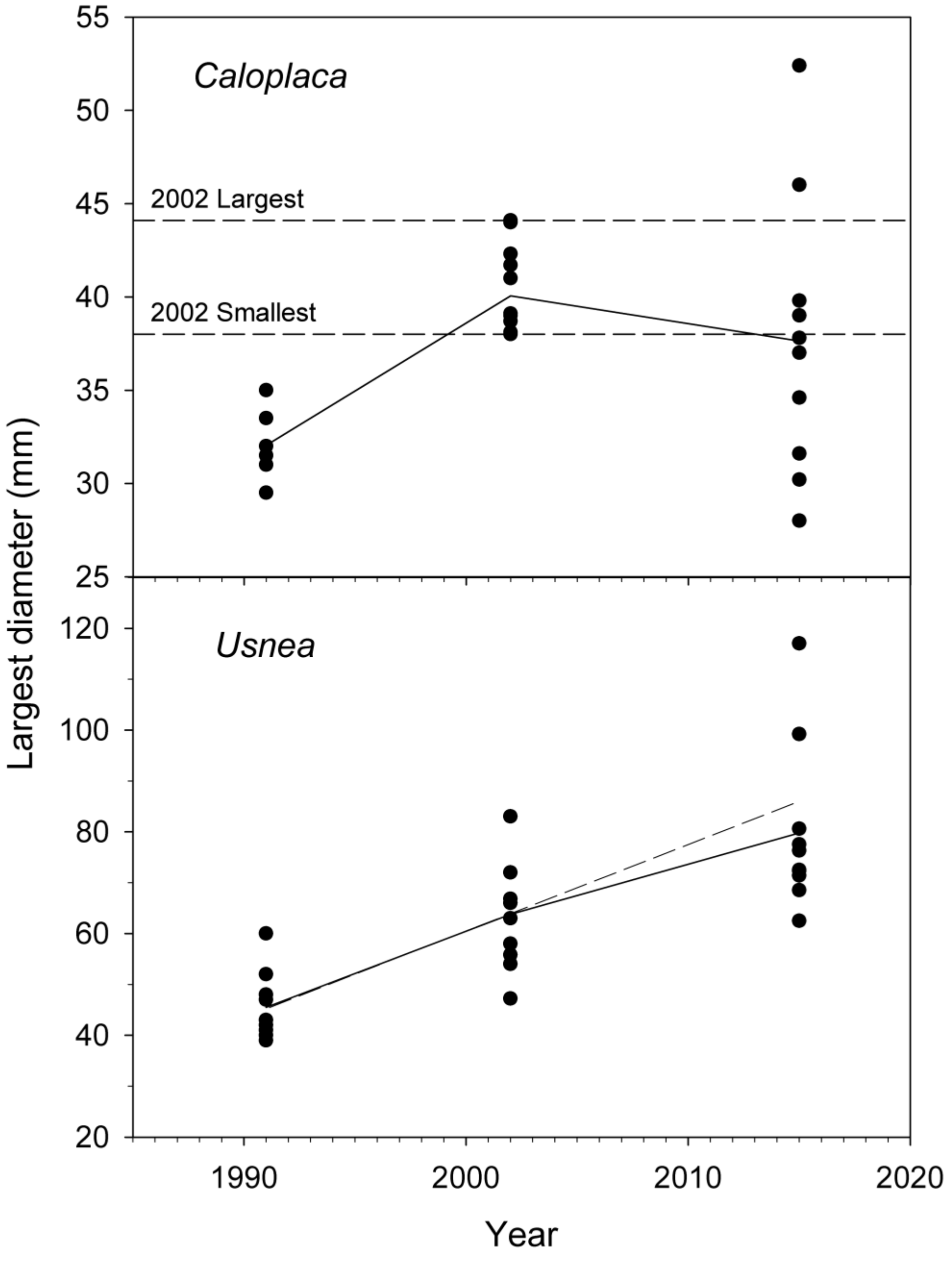

Supplementary Fig. 4: Relationship between annual growth rate normalized to 100% as the fastest rate and mean summer temperature. Growth rates calculated for the crustose lichens *Acarospora macrocyclos* (●), *Bellemerea* sp. (○), *Buellia latemarginata* (▼), *Caloplaca sublobulata* (△), *Rhizocarpon geographicum*, (■) and the fruticose lichen *Usnea antarctica* (□) from measurements in 1991 (from new surface to 1991, 34 years), 2002 (1991 to 2002, 11 years), and 2015 (2002 to 2015, 13 years) and mean summer (January, February and December) temperatures for the same periods in Bellingshausen Antarctic Base, King George Island.

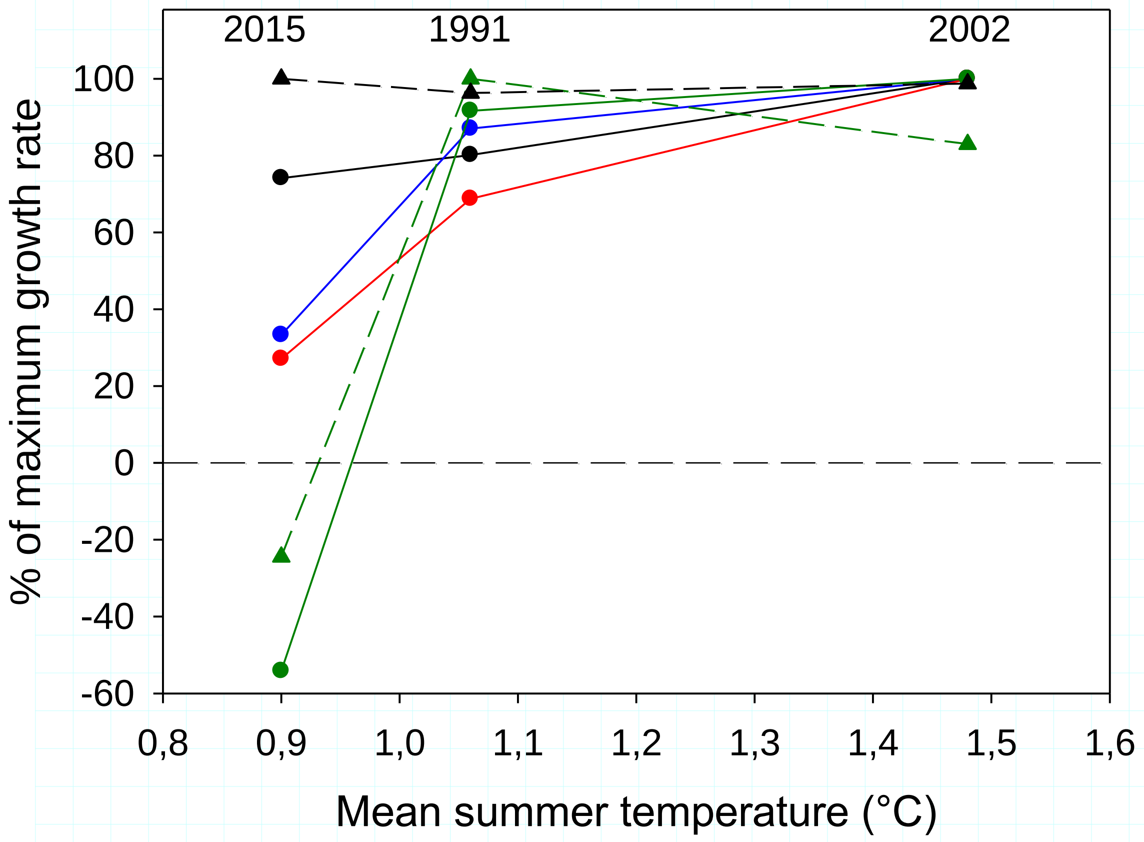

Supplementary Fig. 5 a, b: a: Average air summer temperature in Bellingshausen (black circle. solid line; r^2 = 0.344) and Marsh (white circle. dotted line; r^2 = 0.368) for the whole available series of data. b: Average air summer temperature in Bellingshausen (r^2 = 0.427), Marsh (r^2 = 0.693) and Juan Carlos I (black triangles. dotted line; r^2 = 0.223) since 2002.

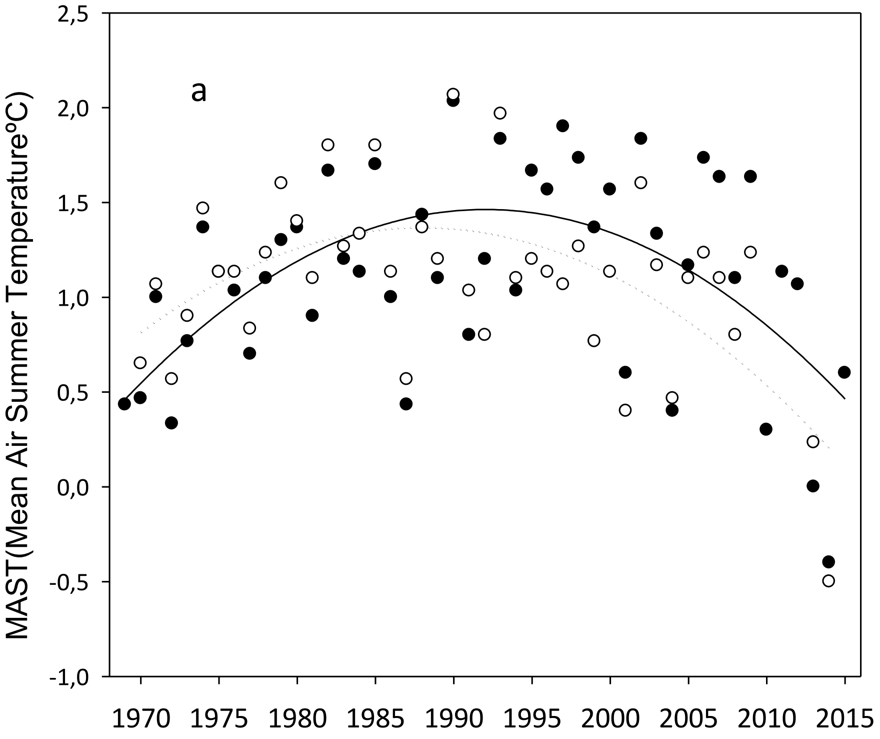

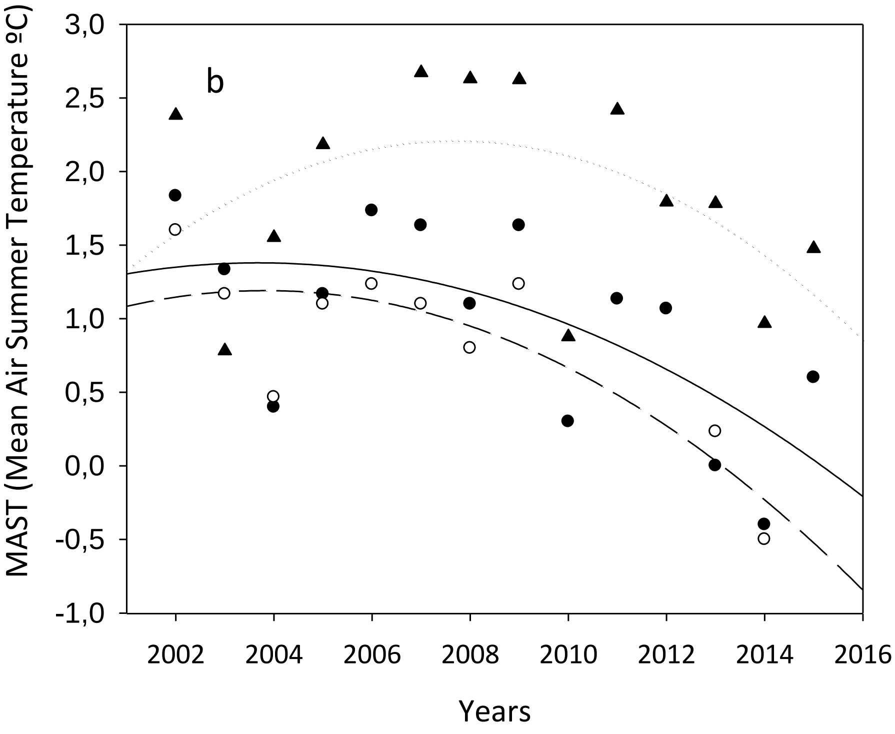

Supplementary Fig. 6: Freezing (black circle, r^2 = 2,85) and thawing (white circle, r^2 = 0,245) indexes in the surroundings of the Spanish Station on Livingston Island during the last 7 years

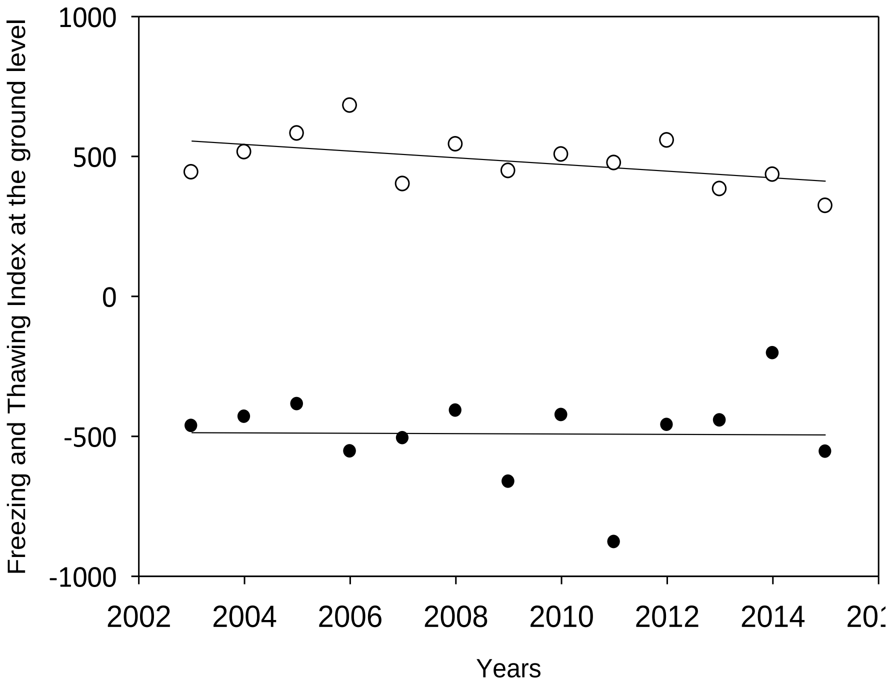

Supplement: Supplementary file 1 — Supplementary Material [file 41598_2017_5989_MOESM1_ESM.pdf]
